# The Critical Biomarkers Identification of Insulin Signaling Involved in Initiating cAMP Signaling Mediated Salivary Secretion in Sjogren Syndrome: Transcriptome Sequencing in NOD Mice Model

**DOI:** 10.1186/s12575-022-00189-5

**Published:** 2022-12-27

**Authors:** Bo Chen, Jiannan Zhou, Tianjiao Mao, Tingting Cao, Shilin Hu, Wenqi Zhang, Xueyang Li, Xiuni Qin, Xintong Liu, Nobumoto Watanabe, Jiang Li

**Affiliations:** 1 Guangdong Engineering Research Center of Oral Restoration and Reconstruction, Affiliated Stomatology Hospital of Guangzhou Medical University, #195 Dongfeng West Road, Guangzhou, 510140 Guangdong China; 2 School of Basic Medicine, Guangzhou Medical University, Guangzhou, Guangdong China; 3 Guangzhou Concord Cancer Center, Guangzhou, Guangdong China; 4 Chemical Biology Research Group, RIKEN Center for Sustainable Resource Science, Wako, Saitama, 351-0198 Japan; 5 Bio-Active Compounds Discovery Unit, RIKEN Center for Sustainable Resource Science, Wako, Saitama, 351-0198 Japan

**Keywords:** Sjogren syndrome (SS), Insulin signaling, cAMP signaling, Salivary secretion, Biomarkers, NOD mice model

## Abstract

**Background:**

Sjogren’s syndrome (SS) is an autoimmune disorder characterized by the destruction of exocrine glands, resulting in dry mouth and eyes. Currently, there is no effective treatment for SS, and the mechanisms associated with inadequate salivary secretion are poorly understood.

**Methods:**

In this study, we used NOD mice model to monitor changes in mice’s salivary secretion and water consumption. Tissue morphology of the submandibular glands was examined by H&E staining, and Immunohistochemical detected the expression of AQP5 (an essential protein in salivary secretion). Global gene expression profiling was performed on submandibular gland tissue of extracted NOD mice model using RNA-seq. Subsequently, a series of bioinformatics analyses of transcriptome sequencing was performed, including differentially expressed genes (DEGs) identification, Gene Ontology (GO) and Kyoto Encyclopedia of Genes and Genomes (KEGG) enrichment analysis, PPI network construction, hub gene identification, and the validity of diagnostic indicators using the dataset GSE40611. Finally, IFN-γ was used to treat the cells, the submandibular gland tissue of NOD mice model was extracted, and RT-qPCR was applied to verify the expression of hub genes.

**Results:**

We found that NOD mice model had reduced salivary secretion and increased water consumption. H&E staining suggests acinar destruction and basement membrane changes in glandular tissue. Immunohistochemistry detects a decrease in AQP5 immunostaining within acinar. In transcriptome sequencing, 42 overlapping DEGs were identified, and hub genes (REN, A2M, SNCA, KLK3, TTR, and AZGP1) were identified as initiating targets for insulin signaling. In addition, insulin signaling and cAMP signaling are potential pathways for regulating salivary secretion and constructing a regulatory relationship between target-cAMP signaling-salivary secretion.

**Conclusion:**

The new potential targets and signal axes for regulating salivary secretion provide a strategy for SS therapy in a clinical setting.

**Supplementary Information:**

The online version contains supplementary material available at 10.1186/s12575-022-00189-5.

## Introduction

Sjogren’s syndrome (SS) is a multi-system rheumatoid inflammatory disease characterized by dry mouth, dry eyes, dry skin, and systemic multi-organ secretion disorders, causing multi-organ system damage and tumors. The high incidence of xerostomia caused by decreased salivary secretion is the acute lesion of the disease and the initiating factor of body system damage [1–5]. Salivary gland secretion dysfunction and structural degeneration lead to SS, which produces not only dry mouth, dry eyes, and systemic dryness, but is also often secondary to systemic diseases such as pulmonary fibrosis, gastritis, liver cirrhosis, pancreatitis, renal insufficiency, osteoarthritis, and neurological disorders, often accompanied by malignant tumors such as thyroid cancer, oral cancer, thymic cancer, and gastric cancer, significantly lymphoma’s risk is increased by more than 40 times [6, 7].

SS prevalence varies among populations ranging from 0.1 to 0.72%, a common rheumatic disease [8, 9]. It is estimated that SS is about 10 times more frequent in women than in men [10]. SS may extend from autoimmune endocrinopathy to affect a variety of extraducular manifestations such as muscle, lungs, blood, and skin [11]. Disease increases mortality compared to systemic lupus erythematosus [12]. Unfortunately, all therapies tested to date have proven ineffective in reversing the course of SS, and research specific to SS in the field of rheumatology is relatively lacking. Therefore, SS significantly harms human health, and an in-depth analysis of its mechanism is of great scientific significance.

Despite significant differences in immune defense mechanisms, evolutionary distance, and living environment, mouse models have been widely used in biomedical research to avoid ethical challenges in human research [13–15]. The non-obese diabetic strain (NOD), a spontaneous autoimmune disease mouse, is a good mouse model for studying SS [16–19]. Salivary flow rate and relative protein concentration in saliva were reduced compared to control mice [20]. Compared with C57BL/6 mice, tear secretion, conjunctival goblet cells, and lymphocyte infiltration were changed [21]. Transcriptome sequencing is a second-generation sequencing platform to obtain almost quickly and comprehensively all transcripts and gene sequences of a specific cell or tissue of a species in a particular state, which can be used to study gene expression, function, structure, alternative splicing, and new transcript prediction. At present, transcriptome sequencing has been widely used in basic research, clinical diagnosis, and drug discovery [22–25].

Here, NOD mice models were selected for animal-level studies to characterize the clinical features of SS. ICR mice were chosen as controls to monitor salivary secretion and water consumption dynamically. The submandibular gland tissues of mice are dissected and extracted to assess morphological changes of the submandibular gland tissues by H&E staining. In contrast, the expression of AQP5, an essential protein secreted by saliva, is evaluated by Immunohistochemical analysis. We obtained the expected experimental results from animal experiments: NOD mice model showed reduced salivary secretion and increased water consumption, H&E staining suggested acinar destruction and changes in the basement membrane, and Immunohistochemical detected a decrease in AQP5 staining in the cell membrane of the acinus. The experimental results also suggest symptoms of xerostomia.

To investigate the regulatory mechanism of salivary secretion in SS, we performed transcriptome sequencing analysis of submandibular gland tissue from extracted NOD mice model using RNA-seq. Subsequently, a series of bioinformatics analyses were performed on RNA-seq, and hub genes (REN, A2M, SNCA, KLK3, TTR, and AZGP1) were identified as the initiating targets of insulin signaling. The effectiveness of the targets was confirmed by analysis and in vitro experiments. In addition, insulin signaling and cAMP signaling are potential pathways for regulating salivary secretion and constructing a regulatory relationship between target-cAMP signaling-salivary secretion.

In conclusion, this study explores a promising novel targeting molecule for predicting SS development and analyzes that insulin signaling and cAMP signaling may be potential pathways for regulating salivary secretion. Hub genes (REN, A2M, SNCA, KLK3, TTR, and AZGP1) serve as initiating targets for insulin signaling and construct a regulatory relationship between target-cAMP signaling-salivary secretion. In SS development, targeting these hub genes may represent a novel strategy for therapeutic interventions in SS patients.

## Materials and Methods

### Animal Study

Female ICR and NOD mice models (6 weeks old, 16-20 g) were purchased from Gempharmatech Co., Ltd. (Jiangsu, China). At a constant temperature of 25 ± 1 °C, all mice freely acquire food and water for 12/12 h in a light/dark cycle. ICR mice and NOD mice, four each, dynamically monitored the indicators of mice. At the observation endpoint, mice were anesthetized to remove submandibular gland tissue for H&E staining and immunohistochemical assays. All procedures involving animals were conducted in accordance with the ethical standards of the Institutional Animal Care and Use Committee of Guangzhou Medical University (approval No. GY2022-119, approved on June 26, 2022).

### Salivary Flow and Water Consumption Assessment

Previously described methods measured salivary secretion [26]. Mice fasted overnight before saliva was collected. Mice were routinely anesthetized with sodium pentobarbital (40 mg/kg) intraperitoneally, and pilocarpine (1.0 mg/kg) was injected subcutaneously. Saliva was collected for 10 minutes: a glass capillary was placed on the side of the mouth under the tongue and held steadily during a 10-min period to collect saliva into a tube. The weight difference in the tubes before and after saliva collection was calculated. The salivary secretion index was calculated by the increase (ug)/body weight (g). The water consumption of NOD and ICR mice for 18 days was recorded. Each mouse’s average water consumption index was calculated as water consumption (ml)/body weight (g), and the changes among mice were observed.

### Hematoxylin and Eosin Staining

Hematoxylin and Eosin (H&E) staining is used to observe general histopathology. Use xylene to dewax submandibular gland sections (5 μM). Gradient ethanol is then used to hydrate the submandibular gland sections. Next, stain the submandibular glands with H&E reagent (Wuhan Servicebio Technology Co. LTD). The tissue morphology of the submandibular glands is examined using light microscopy.

### Immunohistochemical Assays

Paraffin-embedded tissue sections were prepared as 5 μM tissue sections. Tissue sections were then dewaxed in xylene and hydrated in an ethanol gradient. After rinsing with distilled water, a hydrogen oxide solution blocked endogenous peroxidase activity. Tissue sections were incubated with primary antibody AQP5 (1:200) overnight at 4 °C. Tissue sections were then incubated with goat anti-rabbit IgG antibody for 60 min at room temperature. Sections were dehydrated in graded ethanol series and sealed with neutral resin. Finally, the positively stained cells were observed under a light microscope.

### Transcriptome Sequencing and Microarray Data

Submandibular gland tissue from ICR and NOD mice models was extracted, and total RNA was extracted from fresh tissue using Trizol reagent, followed by strict quality control of RNA samples. mRNA with polyA tail is enriched by Oligo (dT) magnetic beads, and the obtained mRNA is randomly interrupted with divalent cations in the NEB Fragmentation Buffer. The library is built according to the NEB standard or strand-specific library building method. Qubit2.0 Fluorometer was used for preliminary quantification, Agilent 2100 bioanalyzer was used to detect the insert size of the library, and qPCR was used to quantify the effective concentration of the library accurately. Based on the HiSeq platform, all mRNAs transcribed from tissues were sequenced, and the sequencing experiment was constructed using the Illumina TruseqTM RNA sample prep kit method. In addition, we downloaded the gene expression profile data GSE40611 [27] from the GEO (http://www.ncbi.nlm.nih.gov/geo/) database as a reference for comparison with our RNA-seq results.

### Hierarchical Clustering Analysis

Bidirectional hierarchical cluster analysis (BHCA) was performed using the “Pheatmap” package in R (v1.0.2, http://cran.r-project.org/web/packages/pheatmap/). Based on the Euclidean distance between expression values, BHCA separately clustered DEGs with similar expression patterns.

### Identification of Differentially Expressed Genes

Raw data were processed using the robust multi-array mean (RMA) algorithm in the “Affy” package in the R language (http://cran.r-project.org/), including background correction, normalization, and probe summarization. Statistically significant differentially expressed genes (DEGs) were mined based on the difference in expression values between NOD and ICR samples using linear models from the “LIMMA” package in R language. Volcano plots were generated using the “ggplot2” package in the R language, and Benjamini-Hochberg’s method was used to control for the false discovery rate (FDR). “P-value < 0.05 and |log2FC| >1” were critical values for screening DEGs.

### Functional and Pathway Enrichment Analyses

DAVID (http://david.abcc.ncifcrf.gov/) is an annotation, visualization, and synthesis discovery database that is an online tool for classifying gene function and evaluating the biological function of genes [28]. GO and KEGG enrichment analysis was performed using the DAVID database to study the role of DEGs. *P* < 0.05 was taken as a statistically significant cut-off point.

### PPI Network Construction and Module Analysis

STRING (v11.5, https://string-db.org) is an online tool for retrieving interacting genes and proteins and entering genes into a database to build interaction networks [29]. In this study, genes with a total score of > 0.4 were selected for network construction. In addition, Cytoscape (v3.8.2, https://cytoscape.org) is used to visualize the networks [30]. Finally, the hub modules in the network are identified by the MCODE plug-in and the degree plug-in in cytoHumba for screening hub genes.

### Predictive Value of Biomarkers

ROC analysis is performed by the “pROC” package of the R language to predict the diagnostic validity of biomarkers. The area under the ROC curve value (AUC) was used to determine the sensitivity of the pSS in the dataset to the diagnosis of the control sample.

### Chemicals and Reagents

The A253 cells were obtained from Zhejiang Meisen Cell Biotechnology Co., Ltd. Fetal bovine serum (Ocamar Technologies Co., Ltd., Shanghai, China). 1640 medium (Ocamar Technologies Co., Ltd., Shanghai, China). Total RNA extraction kit (Shanghai Hengyuan biochemical reagent Co., Ltd., China), IFN-γ (Guangzhou Ruiqian Biotechnology Co., Ltd., China), anti-rabbit monoclonal AQP5 antibody (Abcam, UK, 1:200). Anti-rabbit secondary antibody (Proteintech, China, 1:5000). Hematoxylin and Eosin Staining kit (Wuhan Servicebio Technology Co. Ltd., China). RT-qPCR kit (Invitrogen, USA). RT-qPCR primers (Sangon Biotech, Shanghai, China).

### Cell Culture and Handling

A253 cells were cultured in RPMI 1640 medium containing 10% fetal bovine serum, and the cells were cultured in an incubator at 37 °C, 5% CO2, and saturated humidity. The experiments used cells in the logarithmic growth phase.

Take the cell suspension containing 4 × 10^5^ cells/ml, inoculate it in a 6 cm dish, and use RPMI 1640 medium containing glutamine. Treatment was performed after the cells had adhered. Cells were treated with 100 nM IFN-γ. After 48 h, cells were collected to extract RNA, and the mRNA level of the target gene was detected by RT-qPCR.

### Real-Time Quantitative PCR

Extract total RNA using kit reagents using the GoScript™ Reverse Transcription System Kit (Promega, UK). Use SYBR premix Ex Taq™II (TaKaRa Clontech) following the manufacturer’s instructions, post-transcriptional cDNA for qPCR. Analysis was performed using the 2(−ΔΔCt) method. Detect the gene of interest using primers (Table 1). Analysis using the ΔΔCt method with the gene of interest mRNA = 2-ΔΔCt.

**Table 1:** The primer sequences of qPCR

Primer Name	Forward primer (5′–3′)	Reverse primer (5′–3′)
REN	AGAATGCCCTCAATCCGAGAAAGC	TGTTGCCAAGTGTCAGCCTCTTC
A2M	CACTGTGTCGCCTTCGCTGTC	TTCTTGGGTTGGTCCTTTCACTTGG
SNCA	GTGGCAACAGTGGCTGAGAAGAC	TCTGGGCTACTGCTGTCACACC
KLK3	TCCCACACCCGCTCTACGATATG	GTCCATGACCTTCACAGCATCCG
TTR	CTTACTGGAAGGCACTTGGCATCTC	ACAGCCGTGGTGGAATAGGAGTAG
AZGP1	GGCTCACTCAATGACCTCCAGTTC	CTGCTTCCAATCCTCCATTCCTTCC
β-actin	ATCACTATTGGCAACGAGCGGTTC	CAGCACTGTGTTGGCATAGAGGTC

### Statistical Analysis

Statistical analysis was performed using GraphPad 9. Data are presented as mean ± standard deviation (X ± SD). The data between the two groups were compared using the t-test method. *p* < 0.05 was considered statistically significant.

## Results

### Decreased Salivary Secretion and Increased Water Consumption Rates Were Observed in NOD Mice Model

**Fig. 1:**
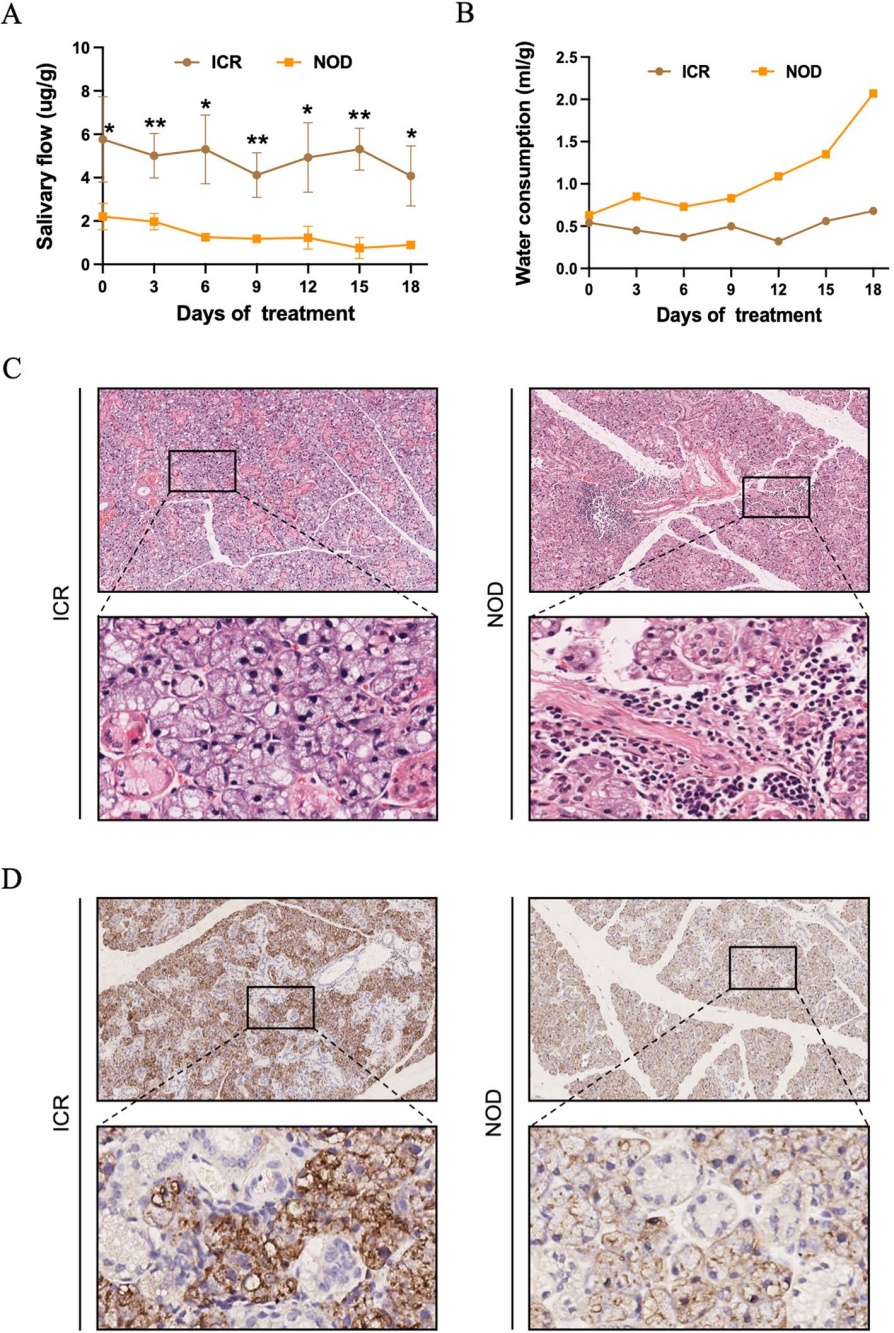
Results of NOD mice model measuring salivary secretion-related indicators. **A** Monitoring of salivary secretion index between NOD and ICR mice. **B** Measurement of water consumption between NOD and ICR mice. **C** Histological images (H&E stained) of mice submandibular gland tissues. **D** Immunohistochemistry images of AQP5 of mice submandibular gland tissues

To evaluate phenotypic characteristics of SS, we compared between NOD mice and ICR mice. The salivary secretion of the mice was monitored at 0, 3, 6, 9, 12, 15, and 18 days, respectively, as shown in Fig. 1A. Compared with the ICR mice, the salivary secretion of the NOD mice was significantly reduced, and the salivary secretion index was 0.19-2.83 μg/g. In the statistics of tear secretion in mice, the results also suggested that compared with the ICR mice, the tear secretion of the NOD mice was significantly reduced, and the tear secretion index was 0.04-0.25 μg/g (Supplementary Fig. 1). To further illustrate the results, we dynamically monitored the water consumption of the NOD and ICR mice over 18 days. The results showed no significant change in water consumption in each stage of ICR, and the water consumption index was 0.32-0.68 ml/g. While the water consumption in the NOD mice increased to 3 times that of the ICR mice, the water consumption increased slowly. It remained at a high level during the observation period (Fig. 1B). These results suggest that NOD mice have decreased salivary secretion and increased water consumption, suggesting symptoms of xerostomia.

### Acinar Destruction and Decreased AQP5 Expression in the Submandibular Gland of NOD Mice

Since salivary secretion is impaired in NOD mice model, we next investigated the expression of a critical protein AQP5 involved in submandibular gland salivary secretion. The results of H&E staining indicated that, compared with the ICR mice, the histology of the submandibular gland tissue in the NOD mice showed acinar destruction and basement membrane changes (Fig. 1C). Studies have shown that acinar cell apical and basolateral membranes show positive AQP5 labeling in mice [31, 32]. In addition, the expression of AQP5 was also detected in intercalated ducts [33]. As shown in Fig. 1D, AQP5 immunostaining in the ICR mice was distributed on the acinar cell membrane. In the NOD mice, AQP5 immunostaining of the cell membrane and apical membrane of the acinus was severely decreased. These data suggest that acinar destruction and AQP5 expression are reduced in the submandibular gland tissue of NOD mice model.

### Processing of Transcriptome Sequencing

**Fig. 2:**
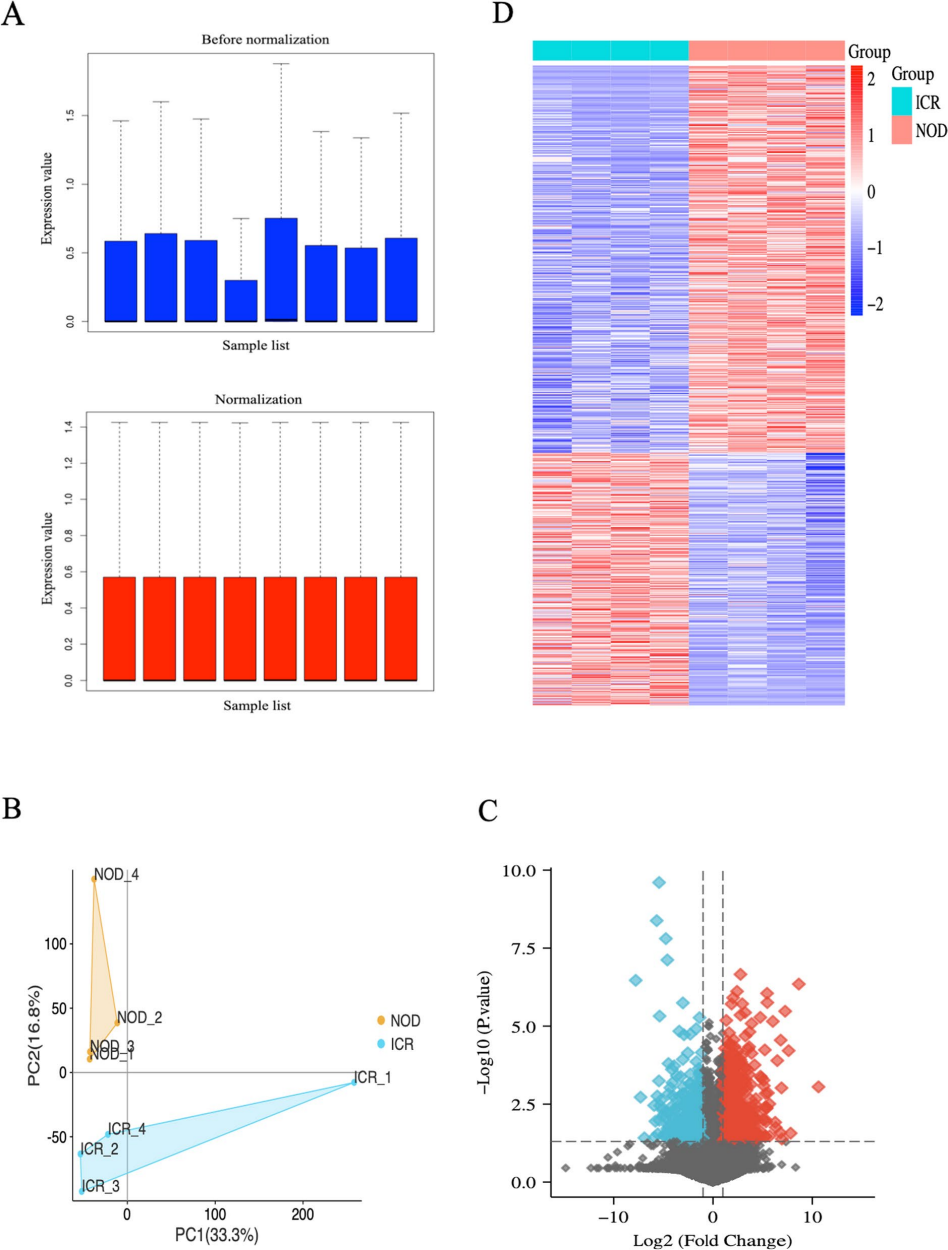
Processing of transcriptome sequencing. **A** Normalization of the transcriptome sequencing data. The black line in each box represents each data group’s median, which determines the degree of normalization of the data through its distribution. The upside is the expression value data before normalization, and the normalized expression value data is the downside. **B** PCA diagram of samples based on expression abundance. **C** The volcano of DEGs. The blue points indicate the screened down-regulated DEGs, the red points indicate the screened up-regulated DEGs, and the black points indicate genes with no significant differences. **D** The heatmap for all DEGs. All DEGs are screened based on *P*-value < 0.05 and |fold change| > 1. DEGs differentially expressed genes; PCA, Principal Component Analysis

To better understand the molecular mechanisms of salivary secretion regulation, we performed transcriptome sequencing analysis of submandibular gland tissues extracted from 6-week-old ICR and NOD mice model using RNA-seq. The dataset was preprocessed using the “Affy” package in R to remove systematically biased genes in the original data. Figure 2A shows gene expression before and after normalization. Based on the normalized data, the NOD and ICR mice were completely distinguished by PCA analysis (Fig. 2B). The DEGs were screened using the “limma” package in the R language, with “P-value < 0.05 and |log2FC|>1” as filter conditions. 834 DEGs were obtained, including 505 up-regulated and 329 down-regulated genes (Fig. 2C). In addition, a heatmap was used to show the expression of all DEGs (Fig. 2D).

### GO and KEGG Enrichment Analysis

**Fig. 3:**
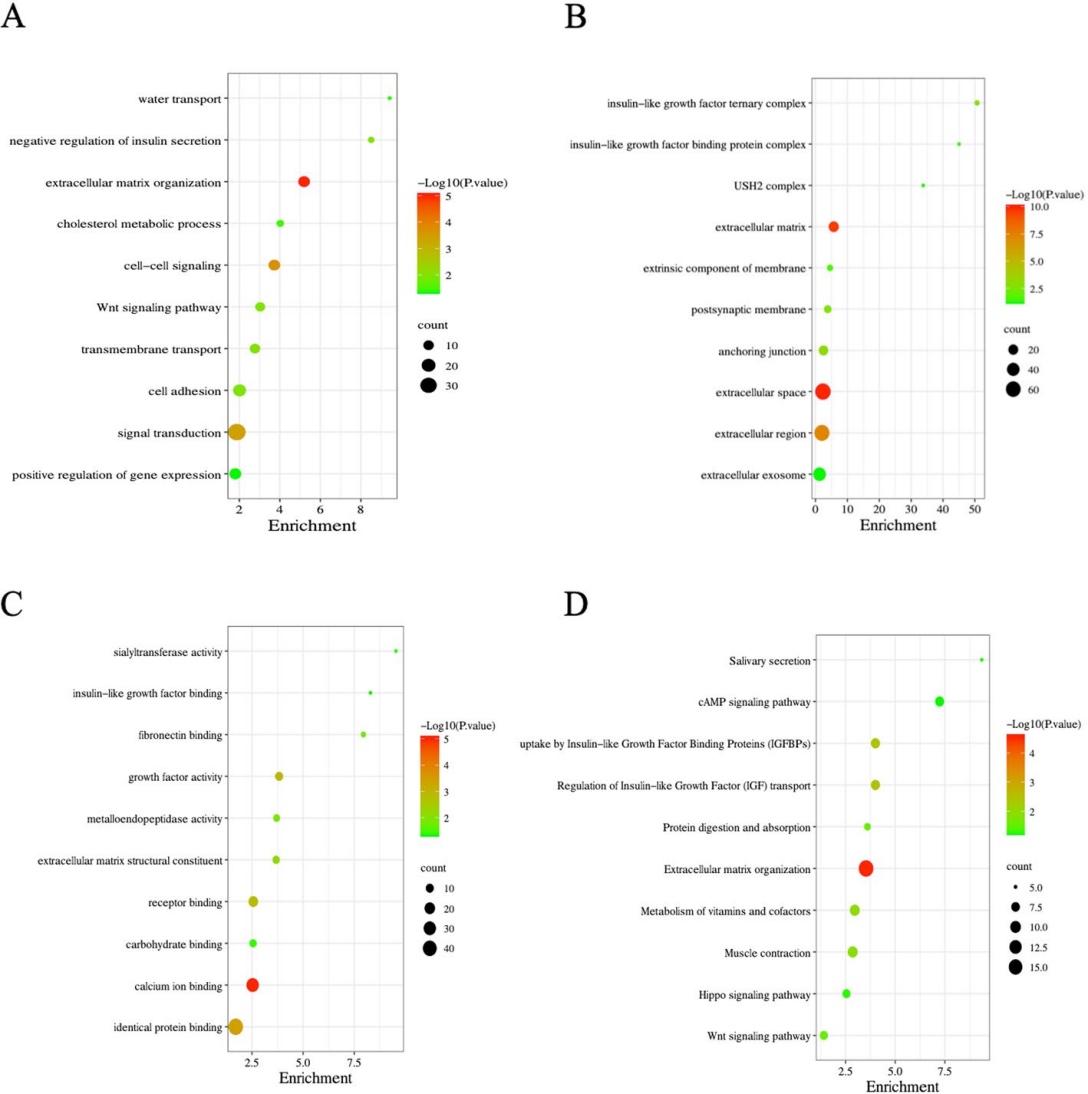
GO and KEGG enrichment analysis. **A-C** GO enrichment analysis of the DEGs. **D** KEGG enrichment analysis of the DEGs. GO, Gene Ontology; KEGG, Kyoto Encyclopedia of Genes and Genomes; DEGs, differentially expressed genes

To further understand the molecular functions and pathways involved in DEGs, we performed an enrichment analysis using the DAVID database. The GO enrichment results showed that these DEGs play critical roles in regulating water transport, insulin secretion, and cell-cell signaling (Fig. 3A-C). Further KEGG enrichment analysis showed that insulin signaling is essential in regulating DEGs. Furthermore, salivary secretion and cAMP signaling significantly enriched KEGG (Fig. 3D).

### Identification of Hub Genes

**Fig. 4:**
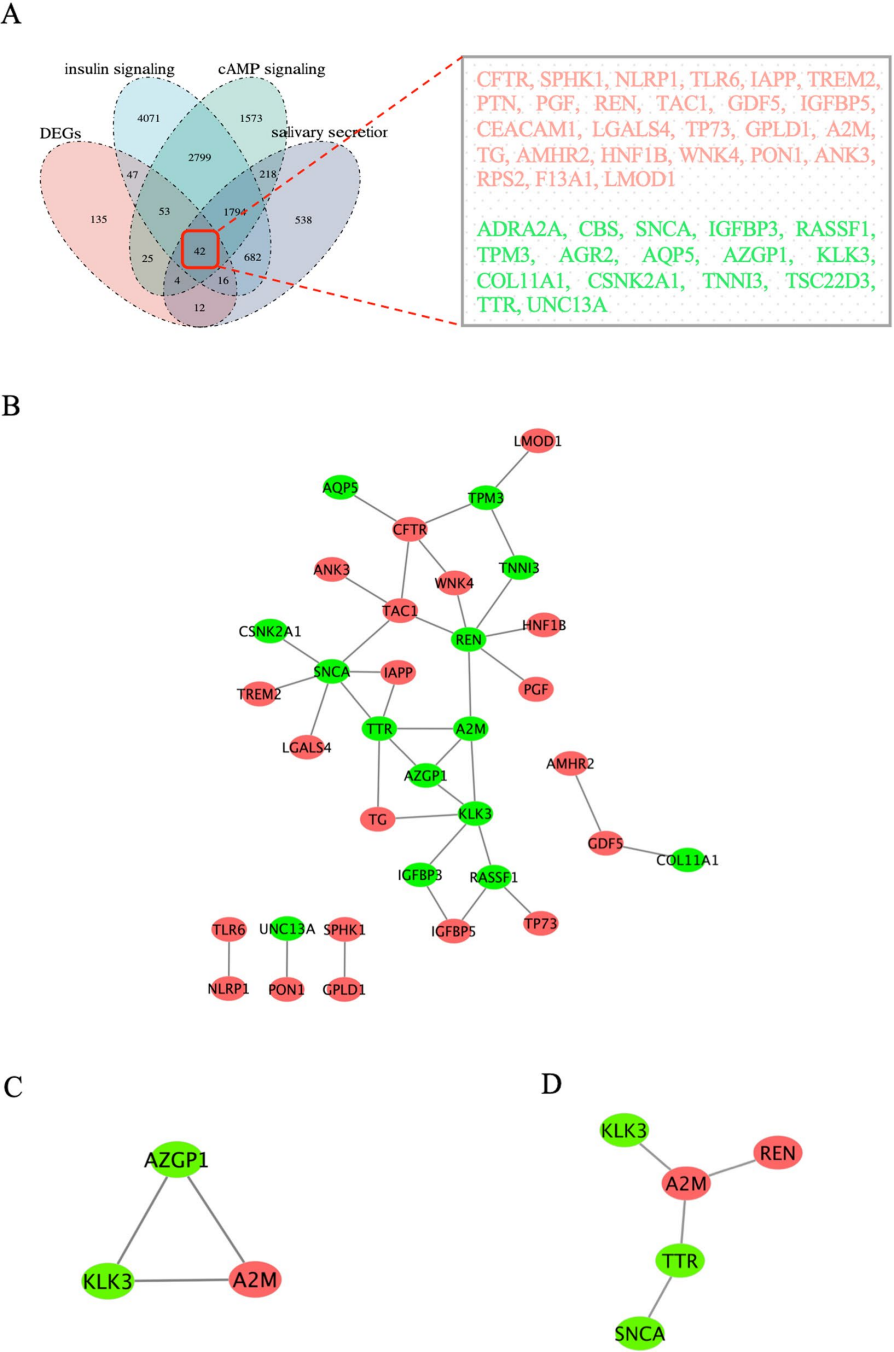
Analysis and screening of hub genes. **A** Venn diagram of 42 overlapped DEGs of insulin signaling, cAMP signaling, salivary secretion, and DEGs. **B** The PPI network of overlapped DEGs was constructed in Cytoscape. **C** MCODE analysis. **D** Degree score sorting. DEGs, differentially expressed genes

First, an integrated analysis of insulin signaling, cAMP signaling, and salivary secretion related genes and DEGs was performed using the Venn tool, and overlapping parts of the four datasets were taken. In our study, 42 overlapping DEGs were identified, including 26 up-regulated DEGs and 16 down-regulated DEGs (Fig. 4A).

To establish protein-protein interactions, we constructed a PPI network for overlapping DEGs using the STRING database, consisting of 42 nodes and 37 edges. After exporting the TSV file, Cytoscape filters out non-interacting proteins and visualizes them (Fig. 4B). In addition, the algorithm of the MCODE plug-in was used for analysis, and the highest-scoring module was selected as the hub module (Fig. 4C). The Degree plug-in identified the network of the top five genes in cytoHubba (Fig. 4D). Finally, we integrated two key networks of genes (REN, A2M, SNCA, KLK3, TTR, and AZGP1) as our hub genes, which are also considered to be initiating regulators of insulin signaling.

### Diagnostic Effectiveness of the Biomarkers

**Fig. 5:**
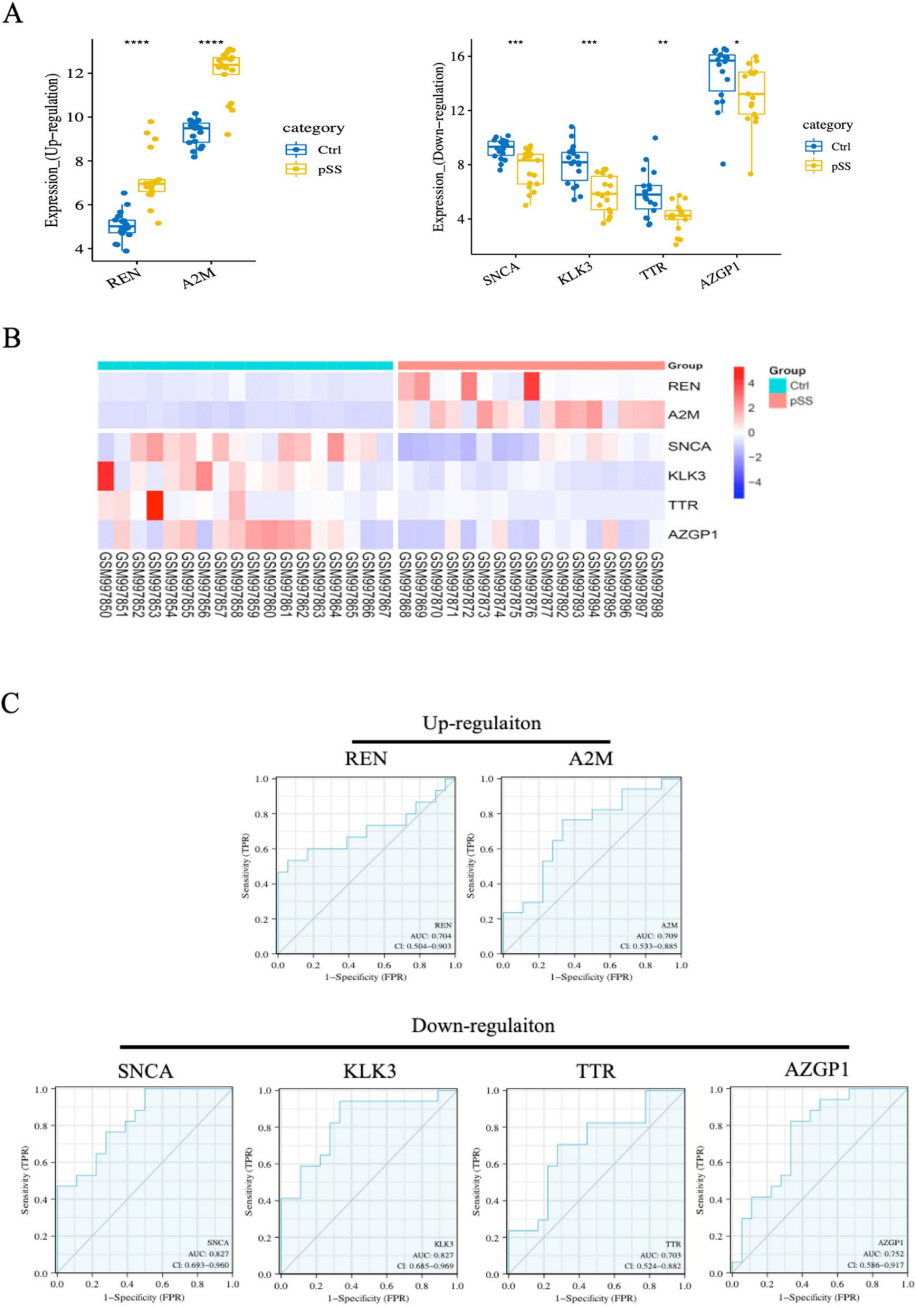
Diagnostic Effectiveness of the Biomarkers. **A** Expression levels of the hub genes between pSS and control samples in GSE40611. **B** The expression heatmap of the hub gene in GSE40611. **C** ROC analysis of hub genes. ROC, Receiver Operator Characteristic Curve

To ensure the reliability of transcriptome sequencing analysis results, we used the dataset GSE40611 from the GEO database to examine the expression of hub genes between the pSS and the control. The number of samples in the pSS group was 17, and the number in the control group was 18. The results showed that the expression levels of hub genes REN and A2M were significantly higher than in the control group (*P* < 0.001). In comparison, the expression levels of the hub genes SNCA, KLK3, TTR, and AZGP1 in the pSS were significantly lower than those in the control group (*P* < 0.05) (Fig. 5A). We further demonstrated the expression of hub genes between pSS and control groups through clustering heatmaps (Fig. 5B). In addition, we performed ROC analysis to detect the diagnostic validity of hub genes as pSS biomarkers, and “AUC > 0.7” is considered to have good sensitivity for pSS diagnosis. As shown in Fig. 5C, in pSS, the AUC values for REN, A2M, SNCA, KLK3, TTR, and AZGP1 are 0.704, 0.709, 0.827, 0.827, 0.703, and 0.752, respectively.

### Construct a Regulatory Relationship between Hub Genes and cAMP Signaling and Salivary Secretion

**Fig. 6:**
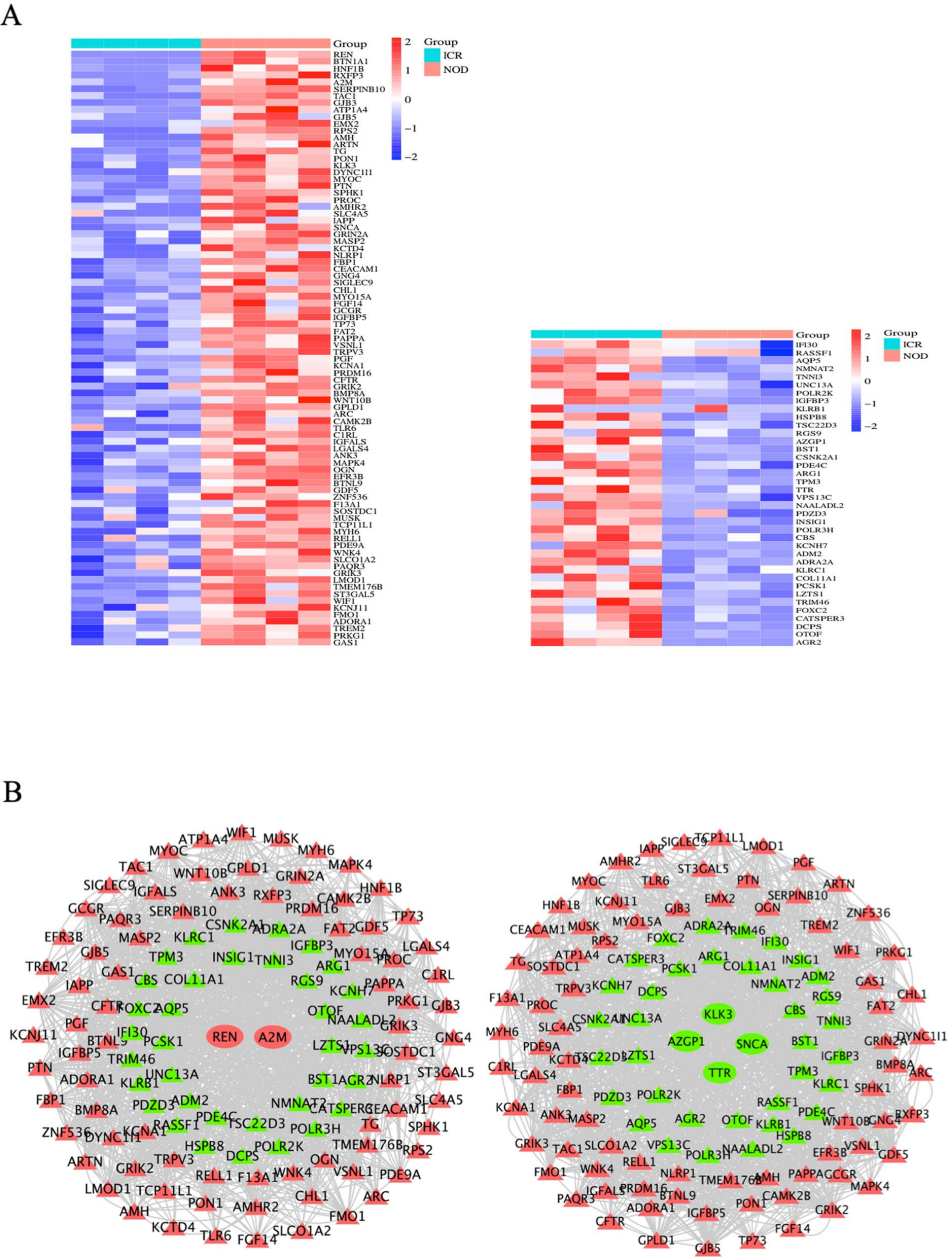
Construct a regulatory relationship between hub genes and cAMP signaling. **A** The cluster heatmap showed the up- and down-regulated overlapping genes (cAMP signaling and DEGs common genes). **B** The regulatory network between the hub and overlapping genes. The red triangle represents the upregulation of common DEGs, the green triangle represents the downregulation of common DEGs, the red circle represents the upregulation of hub genes, and the green circle represents the downregulation of hub genes

**Fig. 7:**
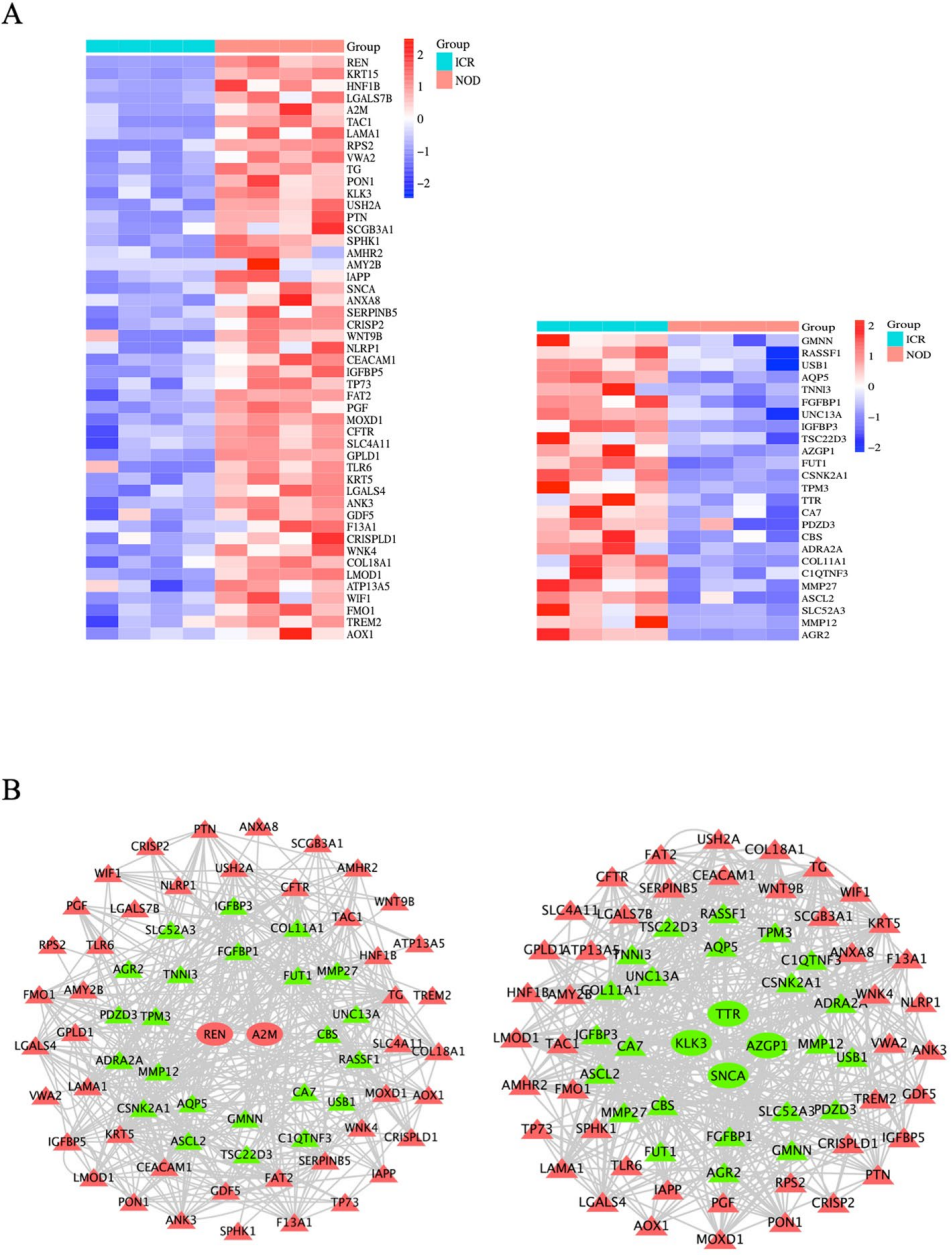
Construct a regulatory relationship between hub genes and salivary secretion. **A** The cluster heatmap showed the up- and down-regulated overlapping genes (salivary secretion and DEGs common genes). **B** The regulatory network between the hub and overlapping genes. The red triangle represents the upregulation of common DEGs, the green triangle represents the downregulation of common DEGs, the red circle represents the upregulation of hub genes, and the green circle represents the downregulation of hub genes

**Fig. 8:**
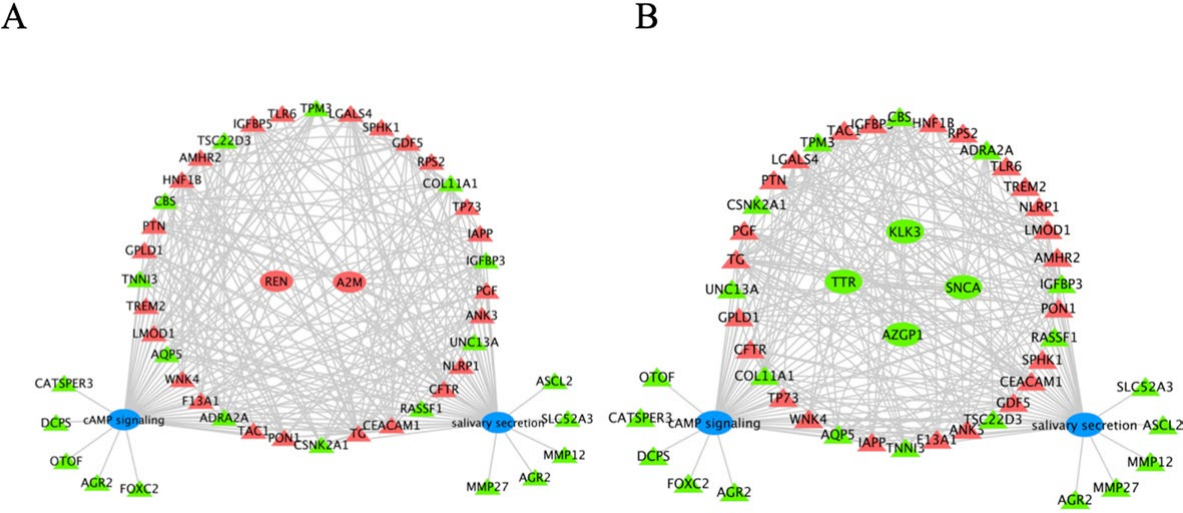
The regulatory relationship between the hub genes, cAMP signaling, and salivary secretion. **A** Construction of regulatory networks between upregulated hub genes and DEGs of cAMP signaling and salivary secretion. **B** Construction of regulatory networks between downregulated hub genes and DEGs of cAMP signaling and salivary secretion. The red triangle represents the upregulation of common DEGs, the green triangle represents the downregulation of common DEGs, the red circle represents the upregulation of hub genes, the green circle represents the downregulation of hub genes, and the blue circle represents the pathway

To investigate the relationship between hub genes and cAMP signaling and salivary secretion, we analyzed them using Venn and Cytoscape. First, we integrated cAMP signaling-related genes and DEGs, took their overlapping parts, and showed the up- and down-regulated overlapping genes through cluster analysis heatmaps (Fig. 6A). Next, we constructed regulatory networks between upregulated hub genes (REN and A2M) and downregulated hub genes (SNCA, KLK3, TTR, and AZGP1) and overlapping genes (Fig. 6B), respectively. From this, we can see that the hub gene plays a crucial role in regulating the cAMP signaling gene. In addition, we also integrated salivary secretion-related genes and DEGs (Fig. 7A) and constructed a regulatory network between hub genes and overlapping genes (Fig. 7B). The results also suggest that hub genes are crucial in regulating salivary secretion genes. Finally, based on these 42 co-expressed genes, we established the regulatory relationship between the hub genes, cAMP signaling, and salivary secretion. CATSPER3, DCPS, OTOF, AGR2, and FOXC2 are the top five-degree genes in cAMP signaling (Fig. 8A), while ASCL2, SLC52A3, MMP12, AGR2, and MMP27 are the top five-degree genes in the salivary secretion (Fig. 8B).

### Experimental Verification of Hub Genes

**Fig. 9:**
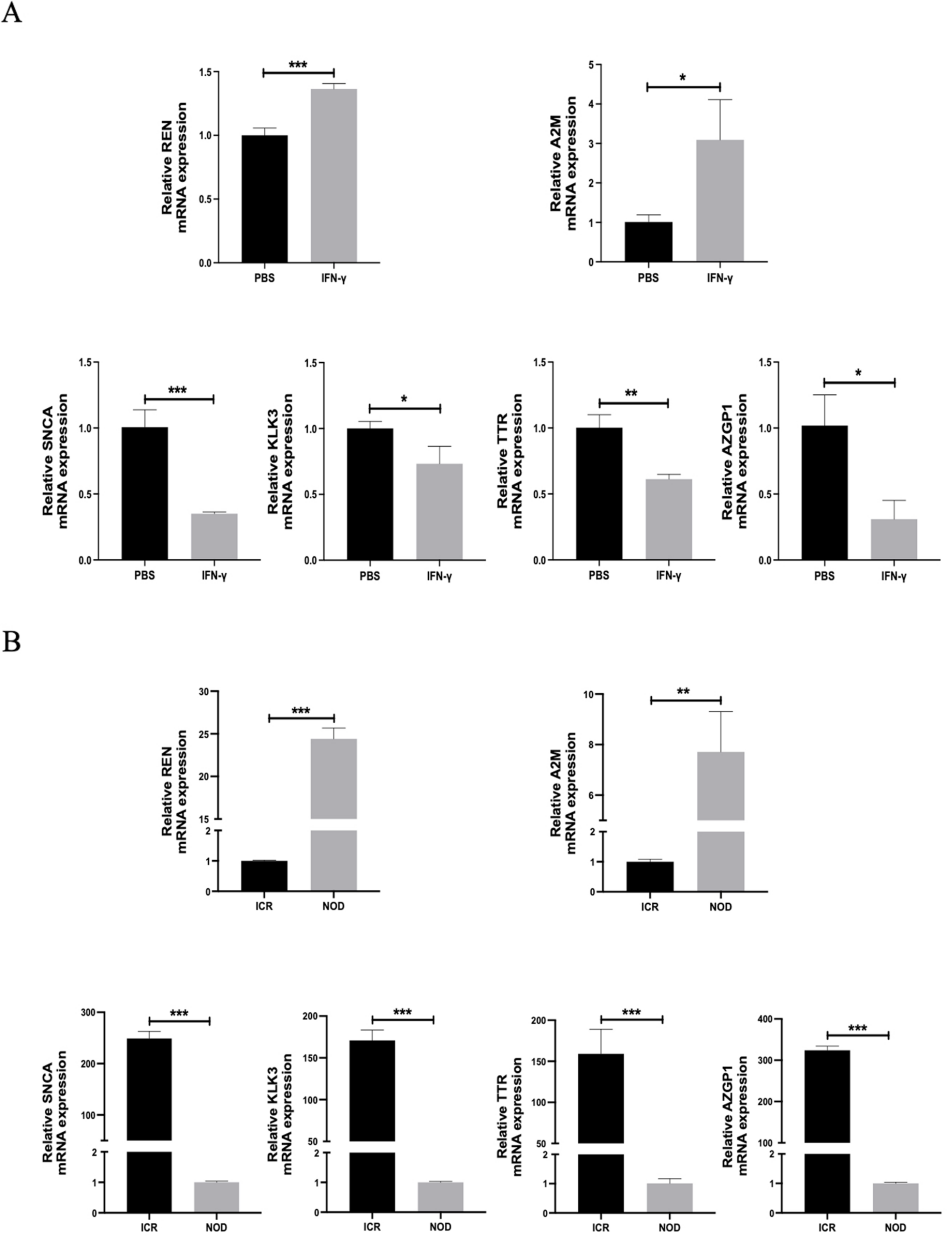
Experimental verification of hub genes. **A** The mRNA levels of REN, A2M, SNCA, KLK3, TTR, and AZGP1 were detected after cells were treated with 100 nM IFN-γ. **B** The mRNA levels of REN, A2M, SNCA, KLK3, TTR, and AZGP1 were detected between NOD and ICR mice submandibular gland tissue

To further validate the results of our transcriptome sequencing analysis, we treated A253 cells with IFN-γ at 100 nM and used PBS as the control group. Changes in the expression of hub genes at the mRNA level are then detected. The results showed that after IFN-γ treatment, the expression of REN and A2M was increased at the mRNA level, while the expression of SNCA, KLK3, TTR, and AZGP1 was decreased at the mRNA level (Fig. 9A). In addition, we selected submandibular gland tissue from NOD and ICR mice, respectively. The results also showed that the expression of hub genes REN and A2M was elevated at the mRNA level. In contrast, the expression of SNCA, KLK3, TTR, and AZGP1 was decreased at the mRNA level (Fig. 9B), which further verified the reliability of the results. These data suggest that the hub gene has some validity in diagnosing SS, which is consistent with the results predicted by sequencing analysis.

## Discussion

Sjogren’s syndrome (SS) is a high-rate, common, and harmful global disease with higher potential morbidity and is an essential menopausal and aging disease [34–36]. The disease is characterized by xerostomia with dysfunction of salivary gland secretion as the initiating factor, which affects the whole body’s health [37–39]. Although SS is a refractory autoimmune disease, the number of studies focusing on essential genes and pathways associated with SS is far less than in systemic lupus erythematosus and rheumatoid arthritis. Molecular and cellular events that occur during SS pathogenesis need to be characterized. The treatment of SS patients is still a clinical challenge, so it is an essential medical topic to study the pathogenesis of the disease and elucidate the regulatory mechanism of abnormal salivation secretion.

In this study, SS was characterized by NOD mice model. We dynamically monitored salivary secretion and water consumption in mice, and NOD mice model experienced decreased salivary secretion and increased water consumption compared with the ICR mice. This result also demonstrates that SS is a disease characterized by dysfunction of salivation. After reviewing all available data on SS mouse models, Ghada et al. noted that the NOD strain is the model that best encapsulates disease characteristics. NOD mice exhibit heterogeneous clinical and laboratory features comparable to SS patients [13]. This also provides good support for our experimental animal research. To further interpret SS at the histological level, we selected submandibular glandular tissue from NOD mice model and applied H&E staining to examine the changes in gland histological morphology. We found that compared with the ICR mice, the submandibular gland tissue of NOD mice model had histology of acinar destruction, basement membrane changes, and lymphocyte infiltration. Studies have shown that SS, in addition to epithelial dysfunction, has several extraglandular manifestations associated with lymphocyte infiltration and B cell overactivity in other organs [40–42]. Numerous studies have shown that aquaporin AQP5 is a crucial target for determining salivary gland secretion and tissue formation [43–45]. Furthermore, the expression of AQP5, an essential protein in salivary secretion, was detected by Immunohistochemical assay. Our study found that the AQP5 immunostaining of the cell and apical membrane of the submandibular gland tissue of NOD mice was severely reduced. These results suggest symptoms of xerostomia.

Next, we performed transcriptome sequencing analysis of extracted submandibular gland tissue from NOD mice model using RNA-seq. Subsequently, differential genes were identified by RNA-seq, and a total of 834 DEGs were identified, including 505 up-regulated genes and 329 down-regulated genes. The biological functions of these DEGs were investigated by DAVID enrichment analysis. The NOD mice significantly enriched water transport, regulation of insulin secretion, and cell-cell signaling. In addition, the KEGG results also suggested that insulin signaling plays an essential role in regulating DEGs, salivary secretion, cAMP signaling, and insulin signaling were the top three significantly enriched results. Insulin signaling is a critical, flexible, and pleiotropic pathway that plays a vital role in maintaining glucose homeostasis. When the glucose homeostasis in the body is disrupted, glucose metabolism decreases, blood glucose rises, and diabetes develops. Studies have shown that diabetes is a disease that causes xerostomia, and there is a significant correlation between the degree of xerostomia and glucose levels in saliva [46]. Hiramatsu et al. have also shown insulin receptor studies that glucose intolerance occurs in Sjogren’s syndrome [47]. These results suggest that insulin signaling may contribute to the critical initiating signal of xerostomia. Numerous studies have shown that cAMP signaling is vital in regulating insulin secretion [48–51]. This provides insights into the regulatory relationship between insulin signaling, cAMP signaling, and salivary secretion.

We used the Venn to analyze insulin signaling, cAMP signaling, and salivary secretion related genes and DEGs, and a total of 42 overlapping genes were identified. Further PPI network construction and modular analysis of these 42 genes identified the hub genes REN, A2M, SNCA, KLK3, TTR, and AZGP1, which are also considered initiating regulators of insulin signaling. By constructing the regulatory network between the hub gene and cAMP signaling and salivary secretion, we found that the hub gene is in a critical position in the network, which provides favorable support for our view. In addition, we downloaded the dataset GSE40611 containing pSS from the GEO database, which further validated our results and ensured the reliability of the sequencing analysis metrics. Finally, to further validate the results of our transcriptome sequencing analysis, we treated cells with IFN-γ and then examined changes in the expression of hub genes at the mRNA level. In addition, we selected the submandibular gland tissues of NOD and ICR mice, respectively, to detect the hub genes. The results indicated that the expressions of hub genes REN and A2M were increased at the mRNA level, while the expressions of SNCA, KLK3, TTR, and AZGP1 were decreased at the mRNA level. These data suggest that hub genes have some validity in diagnosing SS, which is consistent with the results predicted by sequencing analysis.

In interpreting our results, the following limitations need to be carefully discussed: on the one hand, although this study is based on NOD mice model and transcriptome sequencing of submandibular gland material, the sample size is small, and there may be some bias; On the other hand, although we identified the critical targets of insulin signaling and proposed the target-cAMP signaling-salivary secretion regulatory relationship, only these targets were validated at the mRNA level on cells and tissues. Therefore, further precisely designed studies are needed to verify these key targets and regulatory relationships. In conclusion, our analysis reveals previously unknown transcriptional changes in SS and demonstrates the role of transcriptome sequencing-based expression profiling in characterizing disease biomarkers. Our findings may provide new therapeutic targets for SS. Therefore, this analysis can guide future experimental research and clinical translation.

## Supplementary Information

**Additional file 1.**

## Data Availability

All the data supporting the results are included in the article.
