# Supplementary figures and images for: The Critical Biomarkers Identification of Insulin Signaling Involved in Initiating cAMP Signaling Mediated Salivary Secretion in Sjogren Syndrome: Transcriptome Sequencing in NOD Mice Model
Source: Biol Proced Online. 2022 Dec 27;24:26. doi: 10.1186/s12575-022-00189-5 (PMC9793606; doi:10.1186/s12575-022-00189-5)

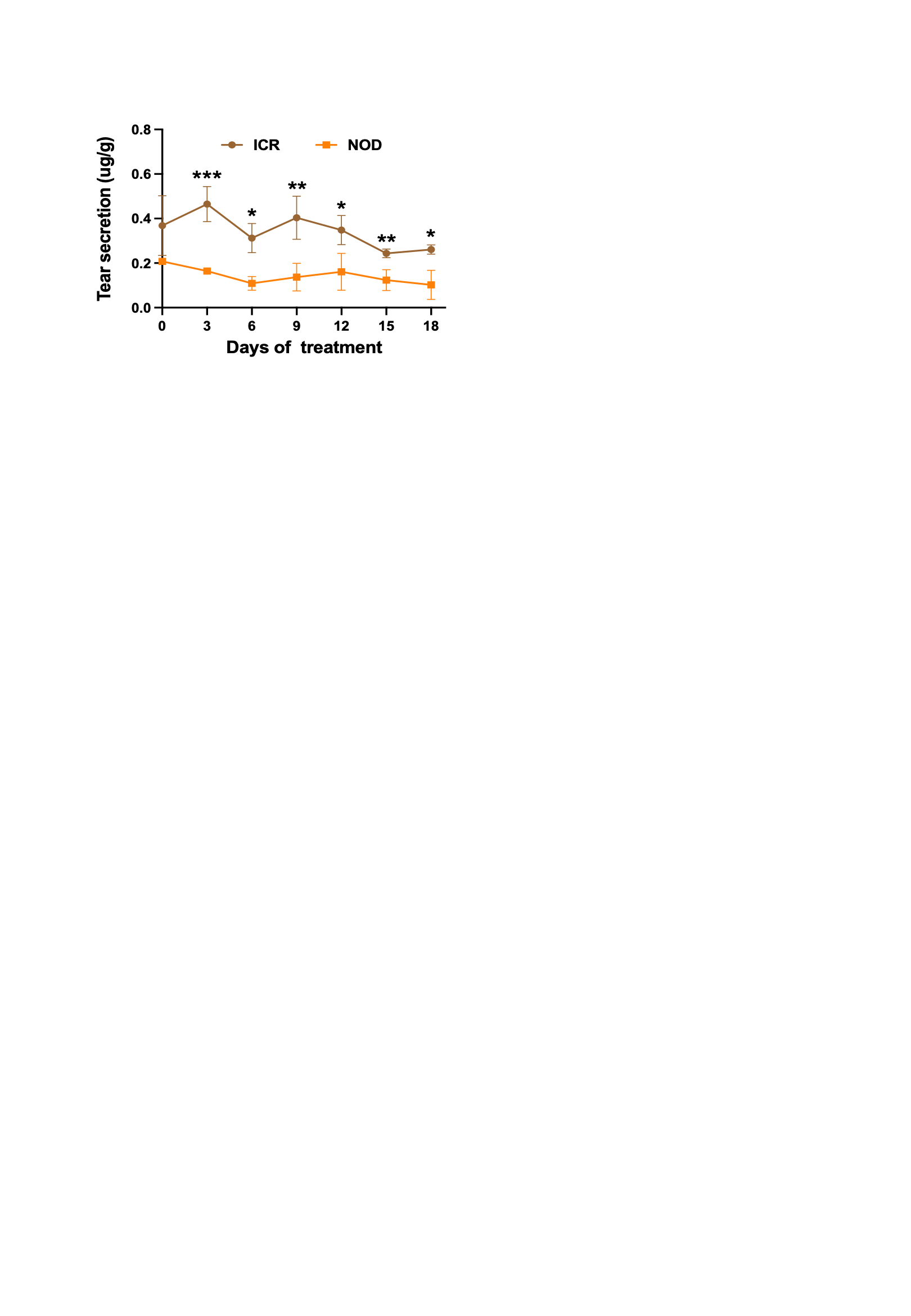

Supplement: Supplementary file 1 — Additional file 1. [file 12575_2022_189_MOESM1_ESM.jpg]
